# Audiogram Estimation by Auditory Brainstem Response with NB CE-Chirp LS stimulus in Normal Hearing Infants

**DOI:** 10.1055/s-0043-1776727

**Published:** 2024-01-24

**Authors:** Diego da Silva Ormundo, Mariana Lopes Fávero, Doris Ruthy Lewis

**Affiliations:** 1 Human Communication and Health Graduate Program, Faculty of Humanities and Health Sciences, Pontifícia Universidade Católica de São Paulo, Universidade de São Paulo, São Paulo, SP, Brazil; 2 Department of Otorhinolaryngology and Phoniatrics, Centro Audição na Criança, Pontifícia Universidade Católica de São Paulo, Universidade de São Paulo, São Paulo, SP, Brazil; 3 Department of Theories and Methods of Speech Language Pathology and Audiology and Physiotherapy, Faculty of Humanities and Health Sciences, Pontifícia Universidade Católica de São Paulo, Universidade de São Paulo, São Paulo, SP, Brazil

**Keywords:** electrophysiology, auditory brainstem response, infants, hearing tests

## Abstract

**Introduction** NB CE-Chirp LS was developed to improve the audiogram estimation by auditory brainstem response (ABR) thresholds during audiological assessment of infants and difficult to test children. However, before we know how the stimulus behaves in several types of hearing loss, it is important we know how the stimulus behaves in normal hearing infants.

**Objective** To describe ABR thresholds with NB CE-Chirp LS stimulus for 500, 1,000, 2,000, and 4,000 Hz, as well as the amplitude and absolute latency for ABR thresholds.

**Methods** Auditory brainstem response thresholds were evaluated with the Eclipse EP25 system. NB CE-Chirp LS was presented using an ER-3A insert earphone. EEG filter was 30 Hz high-pass and 1,500 Hz low-pass. The ABR threshold was defined as the lowest intensity capable of clearly evoke wave V, accompanied by an absent response 5 dB below.

**Results** Eighteen normal hearing infants were evaluated. The mean and standard deviation (SD) of the ABR threshold (dB nHL) were: 23.8 (±4.2); 14.4 (±5.7); 6.0 (±5.0); and 7.0 (±5.9). The mean and SD of the absolute latency (ms) were: 8.86 (±1.12); 9.21 (±0.95); 9.44 (±0.78); and 9.64 (±0.52). The mean amplitude (nV) and SD were: 0.123 (±0.035); 0.127 (±0.039); 0.141 (±0.052); and 0.105 (±0.028), respectively, for 500, 1,000, 2,000 and 4,000 Hz.

**Conclusion** Auditory brainstem response threshold with NB CE-Chirp LS reaches low levels, in special for high frequencies. It provides absolute latencies similar between frequencies with robust amplitude. The results obtained brings to the examiner more confidence in the results registered.

## Introduction

As newborn hearing screening programs have become a standard of care, infants are being referred earlier to pediatric hearing assessment. 1 2 Children with a corrected age up to 6 months do not consistently respond to conventional audiometric techniques, such as visual reinforcement audiometry and conditioned play audiometry. Therefore, it is recommended that audiogram estimation for newborns and young infants are performed with auditory brainstem response (ABR) using frequency specific tones. 3 4 5

Auditory brainstem response threshold in infants, by air and bone conduction, allows the examiner to determine hearing loss configuration, type, and degree. As a result, it is possible to understand the needs of the child for both ears. These measures are important to accurately apply medical and audiological interventions, preventing the underestimation of hearing loss in specific regions of the cochlea, which can occur when a broadband stimulus is used. 3 6 7 8 9 10 11 12 Currently, the most used stimulus in pediatric audiological assessment to determine ABR thresholds for specific frequencies is 500, 1,000, 2,000, and 4,000 Hz tone burst.

Tone burst stimulus triggers neural responses with different absolute latencies for each tested frequency, according to temporal dispersions when the basilar membrane is excited. This occurs because there is no temporal organization of its acoustic spectrum, and the beginning of the presentation for each frequency is located on the zero-latency axis. Thus, the interaction between tone burst and basilar membrane tonotopy results in neural responses with smaller amplitude, poor morphology, shorter absolute latency for high frequencies, and longer latencies for low frequencies. 13

In clinical settings, ABR waveform can bring difficulties in visually identifying peak-picking wave V close to ABR thresholds with tone bursts, specially to younger clinicians, hence longer test times might be necessary, as a greater number of stimuli may be required. Small amplitudes and poor morphologies can also affect the identification of neural responses at intensities close to ABR threshold, making it difficult to accurately estimate the audiogram of an infant. In this sense, CE-Chirp family stimuli were developed in an attempt to improve the ABR recording, optimizing pediatric hearing assessment. 14 15 16

The acoustic spectrum of the Chirp is designed using a delay model, so that low frequency components are presented to the cochlea milliseconds before high frequency components. Thus, the entire basilar membrane is excited at the same time, activating the simultaneous depolarization of inner hair cells and nerve fibers in the auditory pathway. This physiological mechanism triggers neural responses with better synchrony, greater amplitude, better signal-to-noise ratio and morphology, facilitating response detection at low intensities, or close to the behavioral threshold, optimizing test time and audiogram estimation. 17 18

Currently, the CE-Chirp family stimuli used to evoke the ABR is available in the Level Specific (LS) version. For the CE-Chirp LS broadband stimulus, there are variations in the stimulus duration as a function of the intensity level. For the Narrow Band CE-Chirp LS (NB CE-Chirp LS), as frequency-specific stimulus, the variations occur in the latency axis, in the zero-latency reference for 500, 1,000, 2,000, and 4,000 Hz, according to the intensity level. 19 20 Thus, each stimulus was designed differently for each intensity level.

According to the literature, NB CE-Chirp stimulus can be a promising tool in pediatric hearing assessment, as it evokes closer ABR thresholds to the behavioral ones, allowing a better audiogram estimation. 13 21 22 Studies also show that the LS stimulus has advantages in evoking neural responses, especially at loud intensities. 23 24 However, it is necessary to know how the NB CE-Chirp LS stimulus behaves in order to estimate an audiogram for normal hearing infants, and how the evoked neural responses are characterized in terms of amplitude and absolute latency. Therefore, the aim of the present study was to describe ABR thresholds with NB CE-Chirp LS stimulus for 500, 1,000, 2,000, and 4,000 Hz, as well as the amplitude and absolute latency for ABR thresholds.

## Materials and Methods

The present research was carried out at a public clinical hearing healthcare facility, authorized by the Ministry of Health, to perform identification, diagnosis, and early intervention of hearing loss in childhood, in the city of São Paulo, state of São Paulo, Brazil.

The research was approved by the Research Ethics Committee (1.908.319). The parents of the infants signed the Informed Consent Form.

Infants up to 6 months old, who were referred to Newborn Hearing Screening and were assisted at the clinic between January and December 2019, were invited to participate in the study. Only normally hearing infants were selected, according to the following criteria:

### Inclusion

- Normal Transient Evoked Otoacoustic Emissions (TEOAE)
- Presence of waves I, III and V to Click stimulus at 70 dB nHL
- Auditory brainstem response thresholds within normal range for NB CE-Chirp LS stimulus at 500 and 2,000 Hz or 1,000 and 4,000 Hz
- Gestational age ≥ 37 weeks

### Exclusion

- Infants with neurological disorders or head and neck malformations
- Infants with suspected or confirmed syndromes

In case of neurological disorders, the child was referred to a neurologist. For syndromes, a genetic study was provided.

Before the pediatric audiological assessment, the infants had an appointment with an ear, nose, and throat (ENT) doctor, who performed otoscopy and searched for medical history aspects for both the mother and her child.

The TEOAE recording was conducted with Titan – Interacoustics or Institute of Laryngology and Otology (ILO) – Otodynamics. The references for TOAE presence were followed according to each device description and at least for three frequency bands.

Auditory brainstem response was performed with two-channel Eclipse EP25 ABR System. Infants were placed on the parent's lap after their skin was cleansed with Nuprep abrasive paste for proper placement of the electrodes.

Meditrace surface electrodes were positioned with the noninverting in Fz, the ground on the forehead side and the inverting on the right and left mastoids. The test only started when the electrode impedance was < 3 kΩ, and with the infant in natural sleep.

The 500, 1,000, 2,000, and 4,000 Hz NB CE-Chirp LS stimuli were calibrated in accordance with International Organization for Standardization (ISO) standard 389-1: 1998. The stimuli were delivered in only one ear at a time, with insert earphones (ER-3A), and alternating polarity. The repetition rate varied according to each frequency: 37.1/s for 500 Hz; 39.1/s for 1,000 Hz; 45.1/s for 2,000 Hz, and 49.1/s for 4,000 Hz 21 22 .

Neural responses were recorded ipsilaterally and filtered with the 30 Hz high-pass and 1500 Hz low-pass filter, and with ± 40 µV of rejection level. All responses were recorded in a 20-millisecond window. The signal-to-noise ratio (SNR) was estimated by the Fmp technique * , and the residual background noise level (RN) was automatically estimated 25 26 through the equipment's software.

The searching for ABR thresholds started after recording neural responses with clicks at 70 dB nHL in order to analyze neural synchrony. The ABR threshold started at 30 dB nHL for 500 Hz and 20 dB nHL for 1,000, 2,000, and 4,000 Hz, according to the reference values for tone burst stimulus generated in a previous study carried out in our laboratory. 11 The stimulus was decreased in 10 dB steps, until no response was found. Then, a 5 dB step was used until wave V was identified. This recording technique made the threshold more accurate. For all tested intensities, two acquisitions were performed to confirm the response with reproducibility. The minimum intensity level used was 0 dB nHL.

The ABR threshold was defined as the lowest intensity capable of clearly evoking wave V, accompanied by an absent response 5 dB below. The first positive peak, followed by a negative decline (slow negative 10–SN10), recorded after 5 ms, was considered the wave V. It was also necessary to identify a positive SNR and low RN. For the absent response, a flat waveform was considered, with a low RN and a negative SNR.

The SNR was considered positive when the Fmp reached the value of 2.25, corresponding to 95% reliability. When values were below 2.25, the SNR was considered negative. The RN was considered suitable when values were < 25 nV. Recordings were only interrupted after averaging at least 1,500 sweeps.

### Peer-Reviewer

The waveforms and ABR thresholds obtained with NB CE-Chirp LS 500, 1,000, 2,000, and 4,000 Hz were printed and presented to a judge with electrophysiology expertise of at least 10 years. The responses were not marked for wave V. The judge was asked to mark the peak and SN10 of wave V at the ABR threshold, in order to obtain its absolute latency and amplitude.

### Data Analysis

For the hypothesis tests, a significance level of 0.05 was set. The analysis was performed with the aid of RStudio, PASW Statistics for Windows, version18 (SPSS Inc., Chicago, IL, USA), and Minitab (v.18).

## Results

### ABR Thresholds

It was not possible to collect the same data for all frequencies in both ears, since the infants woke up during the test. Eighteen infants were evaluated, with a mean age of 54.6 days (±28.6) and a mean gestational age of 39.6 weeks (±1.2). Nine subjects (50%) were boys and 9 were girls (50%). At 500 Hz, 17 ears were evaluated, 9 on the right side, and 8 on the left side. At 1,000 Hz, 18 ears were evaluated, 10 on the right side, and 8 on the left side. At 2,000 and 4,000 Hz, 20 ears were evaluated, 10 on each side.

Table 1 presents the descriptive summary of ABR thresholds at each frequency. At 500 Hz, there was a statistically significant difference between ears ( *p* < 0.001). The difference was of ∼ 5 dB. Therefore, the results of 2 ears were analyzed in conjunction, with the mean ABR threshold at 500 Hz being 23.8 (±4.2) dB nHL. At 1,000 Hz, the mean ABR threshold was 14.4 (±5.7) dB nHL, and there was no statistic difference between ears ( *p* = 0.079). At 2,000 Hz, the mean ABR threshold was 6.0 (±5.0) dB nHL, and at 4,000 Hz the mean ABR threshold was 7.0 (±5.9) dB nHL, with no statistic difference between the ears for these 2 frequencies, respectively ( *p* = 0.104) and ( *p* = 0.095).

**Table 1:** Descriptive summary of ABR Thresholds (dB nHL) at frequencies of 500 Hz, 1,000 Hz, 2,000 Hz, and 4,000 Hz to right, left, and total ears

Frequencies (Hz)	Ears	N	Mean	SD	*p-value*
500	R	9	26.1	4.2	**< 0.001**
	L	8	21.3	2.3	
	Total	17	23.8	4.2	
1,000	R	10	15.5	6.0	0.079
	L	8	13.1	5.3	
	Total	18	14.4	5.7	
2,000	R	10	6.5	3.4	0.104
	L	10	5.5	6.4	
	Total	20	6.0	5.0	
4,000	R	10	7.5	5.4	0.095
	L	10	6.5	6.7	
	Total	20	7.0	5.9	

Abbreviations: dB nHL, deciBel normalized Hearing Level; L, Left ear; N, number of ears; R, Right ear; SD, Standard Deviation.

From multiple comparison analysis, it was found that the means of ABR threshold across the four frequencies are not all the same, neither in the right ( *p* < 0.001) nor in the left ear ( *p* < 0.001). The results obtained are displayed in Table 2 and show that the mean ABR threshold in 1,000 Hz is lower than in 500 Hz in both ears. For 2,000 Hz, the ABR threshold is lower than in 1,000 Hz in the right ear. The *p* -value is marginal in the left ear, and there was no difference between the means at 4,000 Hz and 2,000 Hz for both ears.

**Table 2:** Multiple comparisons between ABR Thresholds averages at 500 Hz, 1,000 Hz, 2,000 Hz, and 4,000 Hz frequencies

Ear	Mean ABR Thresholds for Frequencies	Mean Difference	Standard Error	95% Confidence Interval	*p-value*
Right	1,000 - 500	- 11.76	2.72	(- 19.37–- 4.15)	**0.002**
	2,000–1,000	- 7.6	2.58	(- 14.83–- 0.38)	**0.037**
	4,000–2,000	- 0.4	2.58	(- 7.62–6.83)	0.999
Left	1,000 - 500	- 10	2.78	(- 17.87–- 2.13)	0.010
	2,000–1,000	- 6.97	2.56	(- 14.22–0.29)	0.062
	4,000–2,000	- 0.67	2.31	(- 7.20–5.87)	0.991

### Absolute Latency and Amplitude of the ABR Thresholds

A descriptive summary of the absolute latency for the ABR threshold at each evaluated frequency is presented in Table 3 . At 500, 1,000, 2,000, and 4,000 Hz, the means for absolute latency were, respectively, 8.86 (±1.12) ms, 9.21 (±0.95) ms, 9.44 (±0.78) ms, and 9.64 (±0.52) ms. There was no statistic difference between the ears at 500 Hz ( *p* = 0.228), 1,000 Hz ( *p* = 0.400), and 2,000 Hz ( *p* = 0.315). Nevertheless, at 4,000 Hz, there was a statistically significant difference between the ears ( *p* <0.001).

**Table 3:** Descriptive summary of latency ABR Thresholds (ms) at frequencies of 500 Hz, 1,000 Hz, 2,000 Hz, and 4,000 Hz to right, left, and total ears

Frequencies (Hz)	Ears	N	Mean	SD	*p-value*
500	R	9	8.81	0.67	0.228
	L	8	8.93	1.53	
	Total	17	8.86	1.12	
1,000	R	10	9.23	1.00	0.400
	L	8	9.19	0.95	
	Total	18	9.21	0.95	
2,000	R	10	9.38	0.73	0.315
	L	9	9.52	0.88	
	Total	19	9.44	0.78	
4,000	R	10	9.44	0.44	**< 0.001**
	L	10	9.83	0.54	
	Total	20	9.64	0.52	

Abbreviations: L: Left ear; ms: milliseconds; N: number of ears; R: Right ear; SD: standard deviation. .

A descriptive summary of the amplitude for the ABR threshold at each tested frequency is presented in Table 4 . At 500, 1,000, 2,000, and 4,000 Hz, the mean amplitudes were, respectively, 0.123 (±0.035) nV, 0.127 (±0.039) nV, 0.141 (±0.052) nV, and 0.105 (±0.028) nV. There was no statistic difference between the ears at 500 Hz ( *p* = 0.171), 1,000 Hz ( *p* = 0.122), and 2,000 Hz ( *p* = 0.563). However, at 4,000 Hz, there was a statistic difference between the ears ( *p* = 0.002).

**Table 4:** Descriptive summary of amplitude ABR Thresholds (nV) at frequencies of 500 Hz, 1,000 Hz, 2,000 Hz, and 4,000 Hz to right, left, and total ears

Frequencies (Hz)	Ears	N	Mean	SD	*p-value*
500	R	9	0.123	0.021	0.171
	L	8	0.124	0.048	
	Total	17	0.123	0.035	
1,000	R	10	0.120	0.033	0.122
	L	8	0.136	0.045	
	Total	18	0.127	0.039	
2,000	R	10	0.142	0.063	0.563
	L	10	0.139	0.042	
	Total	20	0.141	0.052	
4,000	R	10	0.115	0.028	**0.002**
	L	10	0.095	0.026	
	Total	20	0.105	0.028	

Abbreviations: L, Left ear; N, number of ears; nV, nanovolts; R, Right ear; SD, Standard Deviation.

## Discussion

It is known that ABR threshold with click stimulus does not have carry frequency-specificity information and hearing loss in some parts of the cochlea can be underestimated. Also, it is not possible to fit hearing aids without any information on specific frequencies. Thus, narrow band stimuli such as tone bursts and NB CE-Chirp LS were developed to improve hearing assessment of infants who do not respond to visual reinforcement audiometry or conditioned play audiometry.

The gold standard frequency-specific stimulus used in ABR threshold search has been the tone burst, with frequencies at 500, 1,000, 2,000, and 4,000 Hz. However, NB CE-Chirp LS stimulus was developed to improve audiogram estimation of infants, as well as to optimize testing time.

As can be seen in Table 1 , ABR threshold with NB CE-Chirp LS stimulus behaves like the ABR threshold with tone burst reported in the literature. There is a decrease of ABR thresholds when the frequency increases, in both ears. 9 10 The underlying mechanism may be related to the deceleration that low frequency waves undergo in order to reach the cochlear apex. This happens because of the physiology of the basilar membrane in this region. The pattern of the basilar membrane vibration near the cochlear apex affects the neuronal synchronism, and higher ABR thresholds for low frequencies may happen, regardless of the stimulus used. 27

Another factor that may also be involved in this finding is the physiology of the external ear. The external acoustic meatus tends to amplify low frequencies less, and a higher sound pressure level is necessary to activate the basilar membrane in the apical region of the cochlea. 28 29 30

According to Northern et al. (2005) 31 , the normal necessary range of behavioral threshold to ensure the ideal development of language and auditory skills is up to 15 dB HL. With the NB CE-Chirp LS, we were able to record similar ABR thresholds to behavioral results, especially at high frequencies. At a low frequency, such as 500 Hz, the ABR threshold was very close to the normal range, requiring a minor correction factor.

This high precision in the audiogram estimation with NB CE-Chirp LS stimulus is due to its temporal acoustic organization. Each frequency band of this stimulus presents an acoustic spectrum with temporally organized frequency components, which enables the complete stimulation of specific regions of the basilar membrane. This stimulation mechanism triggers neural responses with greater amplitude, which improves the signal-to-noise ratio and enables the visual identification of wave V at increasingly lower intensity levels. 13 17 18 19 21 22

In pediatric audiology, the best audiogram estimation contributes to a better hearing aid fitting and, thus, it is possible to guarantee the necessary audibility for appropriate development of speech and language skills. 13 21 22 In the present study, although we found encouraging results with the NB CE-Chirp LS to obtain ABR thresholds, we emphasize the importance of studies with methods where both the NB CE-Chirp LS and tone burst can be tested in the same infant, to establish correction factors that accurately estimate the audiogram. As a limitation of the present study, it was not possible to test the infants' hearing with both stimuli, due to their short sleep period.

Regarding latencies, it is possible to note that NB CE-Chirp LS stimulus evoked similar ABR thresholds with absolute latencies across frequencies. This finding differs from the literature in relation to tone burst stimulus, which presents a different absolute latency for each frequency. The latency differences found between these stimuli may be related to the delay time model created for different frequencies and intensities. 18 19 20

Tone burst frequencies are programmed to be delivered from zero latency time. For this reason, due to basilar membrane tonotopy, high frequencies are first encoded in the base of the cochlea and trigger neural responses with a shorter latency time, unlike low frequencies that take longer to reach the cochlear apex. 6 8 10 11

The frequency bands of the NB CE-Chirp LS, in addition to having a temporally organized acoustic spectrum, also present temporal compensation among themselves. There is a different delay time for each frequency band, which varies depending on the specific intensity level. These compensations, therefore, allow frequencies to elapse at the same time to reach the region of the basilar membrane where they will be encoded, triggering neural responses with similar absolute latencies. 19 20 32

In the present study, it is also possible to note that, at 500 Hz in the left ears, the standard deviation and the minimum and maximum values varied in content. This result may be related to the wave V morphology, with a rounded peak, which makes it difficult to precisely define its peak and, consequently, to precisely define the absolute latency.

The robust amplitude values found in the present study are related to the acoustic spectrum of the NB CE-Chirp LS, due to the delay model created. This stimulus simultaneously excites the entire length of a narrow region of the basilar membrane and triggers the synchronous depolarization of a greater number of neuronal fibers corresponding to the stimulated region, generating a neural response with greater amplitude. 13 17 18 19 20 21 22 32

As infants are usually assessed during natural sleep, it is important that the ABR threshold be collected quickly. Thus, the amplitude robustness from neural responses triggered with NB CE-Chirp LS stimulus can optimize testing time. In addition, seeing a wave V with higher amplitude, as well as with the similar latency time between frequencies, can make it easier for less experienced examiners to capture the peak-picking wave V, making the professionals more confident in the responses viewed. 21 22

Although tone burst stimulus has been considered the gold standard for estimating the behavioral threshold of babies, 33 34 NB CE-Chirp LS stimulus has been gaining more space in pediatric hearing assessment, as it allows a better audiogram estimation. In addition, to optimizing test time and facilitating the identification of responses, this stimulus evokes neural responses with a higher signal-to-noise ratio and with a similar absolute latency for different frequencies. However, other studies with subjects of different age groups and with different types, degrees, and configuration of hearing loss must be carried out.

## Conclusion

Auditory brainstem response threshold with NB CE-Chirp LS reaches low levels depending on the frequency and in special for high frequencies such as 2,000 and 4,000 Hz. It provides similar absolute latencies between frequencies with robust amplitude. The results obtained bring to examiners more confidence in the results registered.

## Footnotes

*
*Fmp (frequency multi points) is a statistical analysis in which the software compares the amplitude of the neural response to residual background noise and provides a confidence level for the recorded response.* 25 26
